# Epilepsy‐specific patient‐reported outcome measures of children's health‐related quality of life: A systematic review of measurement properties

**DOI:** 10.1111/epi.16430

**Published:** 2020-01-17

**Authors:** Holly Crudgington, Morwenna Rogers, Hannah Morris, Paul Gringras, Deb K. Pal, Christopher Morris

**Affiliations:** ^1^ Basic and Clinical Neuroscience Department Institute of Psychiatry, Psychology and Neuroscience King's College London London UK; ^2^ University of Exeter Medical School University of Exeter Exeter UK; ^3^ Evelina London Children's Hospital London UK; ^4^ KCL Institute for Women and Children's Health London UK; ^5^ MRC Centre for Neurodevelopmental Disorders King's College London London UK; ^6^ King's College Hospital London UK

**Keywords:** children, epilepsy, paediatric, patient‐reported outcome measures, young people

## Abstract

**Objective:**

To identify and appraise published evidence of the measurement properties for epilepsy‐specific patient‐reported outcome measures (PROMs) of children's health‐related quality of life (HRQoL).

**Methods:**

We searched multiple databases for studies evaluating the measurement properties of English‐language epilepsy‐specific PROMs of children's HRQoL. We assessed the methodological quality using the COnsensus‐based Standards for the selection of health Measurement INstruments (COSMIN) guidance. We extracted data about the content validity, construct validity, internal consistency, test‐retest reliability, proxy reliability, responsiveness, and precision, and assessed the measurement properties with reference to standardized criteria.

**Results:**

We identified 27 papers that evaluated 11 PROMs. Methodological quality was variable. Construct validity, test‐retest reliability, and internal consistency were more commonly assessed. Quality of Life in Childhood Epilepsy (QoLCE) questionnaires are parent‐reported and evaluated more than other PROMs; QoLCE‐55 has good and replicated evidence for structural and construct validity and internal consistency. Health‐Related Quality of Life Measure for Children with Epilepsy (CHEQoL) has both child and parent‐reported versions and good evidence of content, structural, and construct validity.

**Significance:**

This review identified two leading candidate epilepsy‐specific PROMs for measuring health‐related quality of life in children. Establishing evidence of the responsiveness of PROMs is a priority to help the interpretation of meaningful change scores.

**Key points**

- We identified 27 papers that evaluated the measurement properties of 11 epilepsy‐specific PROMs of children's HRQoL.
- PROMs with more evidence of robust measurement properties included the QoLCE‐55, QoLCE‐76 (parent‐only report), and CHEQoL (parent and child reported).
- Evidence of responsiveness is lacking for epilepsy‐specific PROMs of HRQoL in children, which limits understanding of how much change in scores is perceived important and exceeds measurement error.

## 1 INTRODUCTION

Epilepsy is a common, chronic neurological condition that is characterized by a tendency to have recurring seizures.^1^ Epilepsy occurs in people of all ages and affects around 3.2 in 1000 children in Europe.^2^ There are a number of different pediatric epilepsy syndromes of which childhood epilepsy with centrotemporal spikes (CECTS) is the most common.^3^ Epilepsy is associated with a range of cognitive, psychiatric, social, and language issues that can lead to considerable challenges for the child and their family.^4^ Typically, seizures are treated with antiepileptic medications, although the child can often incur unwanted side effects.^4^, ^5^

Health‐related quality of life (HRQoL) is an increasingly important focus for research in childhood epilepsy due to the medical, social, and psychological complications of seizures and antiepileptic medications. A patient‐reported outcome measure (PROM) is a standardized questionnaire that is completed by a patient to measure their perception of their own health, well‐being, and/or HRQoL; there may be proxy versions for carers to complete. Some PROMs are generic and designed for use across all health conditions and others are condition‐specific. PROMs may focus on a singular aspect of health or have several domains that measure the multifaceted dimensions of HRQoL. PROMs are used widely to inform clinical practice such as monitoring a patient's health in national audits of health services and for collecting information on treatment outcomes for clinical trials.^6^, ^7^

A number of reviews have highlighted the condition‐specific and generic PROMs that are available for use in pediatric epilepsy.^8^, ^9^, ^10^, ^11^, ^12^, ^13^, ^14^ For a PROM to be considered robust it needs to meet the standard criteria for measurement properties such as does the PROM measure what it purports to measure and is it understandable by the target population (validity), does the PROM measure this in the population in the same way each time (reliability), does it detect changes accurately without measurement error (precision), and how much change is meaningful to patients and considered clinically important (responsiveness). Initiatives such as COnsensus‐based Standards for the selection of health Measurement INstruments (COSMIN) have been established and recently updated to provide researchers with standardized, evidence‐based resources to appraise the measurement properties of PROMs and a framework for conducting systematic reviews.^15^, ^16^

In this review, our aim was to examine which epilepsy‐specific PROMs of children's HRQoL can be considered robust by finding, appraising, and summarizing evidence from published studies evaluating their measurement properties.

## 2 METHODS

### 2.1 Search strategy

**Figure 1:**
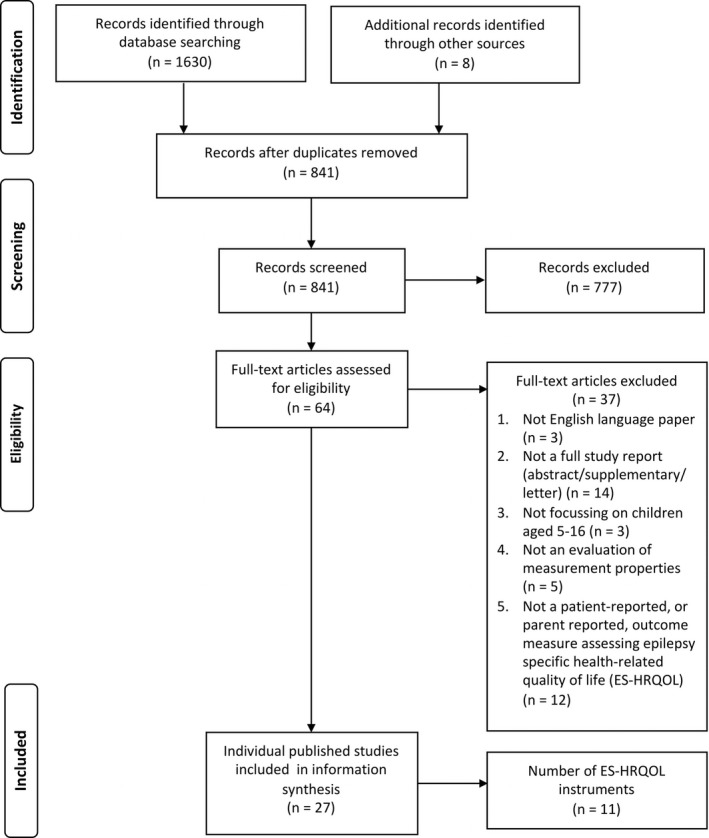
Flowchart illustrating identification and selection of eligible studies

We identified PROMs using structured, systematic review methods described in our protocol.^17^ Our electronic search strategy used the names of known childhood epilepsy questionnaires, identified by an existing systematic review.^9^ In addition, we combined terms for epilepsy with generic terms for PROMs such as questionnaire, measure, tool, and scale. The databases CENTRAL (via the Cochrane Library), MEDLINE (via OvidSp), EMBASE (ViaOvidSp), PsycINFO (via OvidSp), and CINAHL (via EBSCOhost) were searched in May 2018 by MR (Appendix S1). We searched for additional relevant papers in the reference lists of included papers and we undertook forward reference searching via Google Scholar by checking articles that cited the original questionnaire validation articles. We contacted the corresponding author of each included article to confirm that we had not missed any other relevant articles. The search strategy was recorded in a PRISMA flowchart (Figure 1).

### 2.2 Inclusion and exclusion criteria

Articles were selected if they evaluated the measurement properties of epilepsy‐specific PROMs for children (or by parent/caregiver proxy report). We only considered articles published in peer‐reviewed journals. Articles were excluded if (a) the article was not an English‐language paper, or not an evaluation of an English‐language version of the questionnaire, (b) not a full report (eg, conference abstracts were excluded if a paper was not subsequently published), (c) not focusing on children aged 5‐16, unless data on children could be extracted separately, (d) not an evaluation of measurement properties, (e) not a patient‐reported, or parent‐reported, outcome measure assessing epilepsy‐specific Health‐Related Quality of Life (HRQoL).

### 2.3 Study selection

Two reviewers (MR and HM) independently reviewed all titles and abstracts obtained from the literature searches. Any disagreements were arbitrated by a third reviewer (CM).

The full texts of all potentially eligible studies were retrieved. Two reviewers (HC and HM) independently assessed each full text against the exclusion criteria. Disagreements were resolved by discussion between the two reviewers or arbitrated by a third reviewer (CM).

### 2.4 Data extraction

First, descriptive data were extracted from included papers. Second, data were extracted on the measurement properties of the PROMs, which included content validity from qualitative research and/or any theoretical framework, structural validity determined using factor analysis, internal consistency of scales, construct validity evaluated by correlation with other scales, and hypothesis testing to verify scales measure the intended construct, test‐retest reliability, proxy reliability between child and parent, precision, and responsiveness (whether change in scores are considered robust).

### 2.5 Risk of bias analysis

We assessed each included paper for its methodological quality using the COSMIN risk of bias (RoB) checklist for use in the systematic reviews of PROMs.^15^, ^16^ Using the checklist, we assessed the bias of each paper for how the following seven properties had been tested: content validity, structural validity, construct validity, internal consistency, test‐retest reliability, precision, and responsiveness. We rated any measurement property evaluated in each paper using the COSMIN four‐point scale: “very good,” “adequate,” “doubtful,” “inadequate.”

### 2.6 Synthesis of results

We synthesised all evidence of methodological quality of studies and measurement properties of PROMs using standard reference criteria as we have done in other similar reviews.^18^ We interpreted the evidence for each questionnaire into an overall rating of evidence and collated this into a summary appraisal table (Table 4). Two reviewers (HC and CM) appraised the information and agreed a summary rating. We provide a brief succinct narrative synthesis of the findings in the results; fuller details of data extracted with longer narrative are available in the supplementary files (Appendix S2).

## 3 RESULTS

We found 27 studies that evaluated the measurement properties of 11 epilepsy‐specific PROMs of children's HRQoL (Figure 1). Authors of the articles that we contacted did not suggest any further papers that we had not already included. Of the 11 PROMs, QoLCE‐76 has three shortened versions, and the Impact of Childhood Illness (ICI) scale PROM has two prior versions. Collectively, the PROMs are for children aged 3 months to 18 years of age with epilepsy and were developed in the UK, Europe, USA, Canada, and Australia (Tables 1 and 2). The methodological quality of the included studies was variable across a range of measurement properties (Table 3). Construct validity, test‐retest reliability, and internal consistency were more commonly assessed properties across studies; evaluations of responsiveness and precision were lacking (Table 4, Appendix S2).

**Table 1:** Epilepsy‐specific patient reported outcome measures (PROMs)

No.	Instrument version	Author	Purpose	No. of items and domains	Age range	Country/origin	Respondent
1a	Adult's Attitudes to Children with Epilepsy: Visual Analogue Scale	Hoare (1986)^22^	Assess adult's attitudes to children with epilepsy	47 items, 7 domains: Physical consequences of a single fit; Aetiology of epilepsy; Problems for the child at present and in the future; Side effects of drugs; Problems for the child's parents; Social restrictions or the child and his family; Adverse effects of family life	10	Edinburgh, UK	Parent
1b	Modified Impact of Epilepsy Schedule	Hoare (1993)^21^	Assess adult's attitudes to children with epilepsy and the impact on adults	39 items, 3 domains: The medical care and treatment of epilepsy; The child's adjustment and development; Effects on family life	5‐15 y	Edinburgh, UK	Parent
1c	The Impact of Childhood Illness Scale (ICI)	Hoare et al (2000)^19^	Assess the impact of epilepsy/long‐standing childhood illness on QoL on the child and family	30 items, 4 domains: Impact on the child's development and adjustment; impact on the parents; and impact on the family and a combined total score. The instrument is scored on two dimensions: Frequency and Importance.	6‐17 y	Edinburgh, UK	Parent
2	The Hague Restrictions in Childhood Epilepsy Scale (HARCES)	Carpay et al (1997)^24^	Quantify restrictions due to disability in childhood epilepsy	10 items, including 2 global items	4‐16 y	Hague, Rotterdam	Parent
3	Quality of Life in Epilepsy Inventory for Adolescents (QoLIE‐AD‐48)	Cramer et al (1999)^25^	Assess HRQoL in adolescents with epilepsy	48 items, 8 domains: Epilepsy impact; Memory/concentration; Attitudes towards epilepsy; Physical functioning; Stigma; Social support; School behaviour; Health perceptions and a total summary score.	11‐17 y	USA & Canada	Child
4	Quality of Life in Pediatric Epilepsy (QoLPES)	Arunkumar et al (2000)^26^	To assess HRQoL in children with epilepsy	20 items	3 mo‐18 y	USA	Parent & child
5a	Quality of Life in Childhood Epilepsy (QoLCE)	Sabaz et al (2000) & Sabaz et al (2003)^27^, ^28^	To assess HRQoL for children with epilepsy	Australian version: 73 items, 16 subscales, covering 7 domains: Cognition, Physical activities, Social activities, Emotional wellbeing, Behavior, General health, General Quality of Life and a total score USA version: 76 items, 16 subscales, covering 7 domains: Cognition, Physical activities, Social activities, Emotional well‐being, Behavior, General health, General Quality of Life and a total score	4‐18 y	New South Wales, Australia & USA	Parent
5b	QoLCE 55	Goodwin et al (2015)^30^	To assess HRQoL for children with epilepsy, in a shortened version	55 items, 4 domains: Cognitive; Emotional; Social and Physical	4‐18 y	Canada	Parent
5c	QoLCE 16	Goodwin et al (2018)^33^	To assess HRQoL for children with epilepsy, in a shortened version	16 items, 4 domains: Cognitive; Emotional; Social and Physical	4‐18 y	Canada	Parent
5d	G‐QoLCE	Conway et al (2018)^34^	To assess HRQoL for children with epilepsy with one item	1 item	4‐18 y	Canada	Parent
6	Impact of Pediatric Epilepsy Scale (IPES)	Camfield et al (2001)^35^	To assess the influence of epilepsy on the major aspects of the family and child's life	11 items	2‐16 y	Canada	Parent
7	Health‐Related Quality of Life Measure for Children with Epilepsy (CHEQoL‐25)	Ronen et al (2003)^38^	To measure the HRQoL of preadolescent children with epilepsy	25 items, 5 domains: Interpersonal/Social Consequences; Worries and Concerns; Intrapersonal/Emotional Issues; Epilepsy My Secret and Quest for Normality	6‐15 y	Canada	Child and parent
8	DISABKIDS (Epilepsy Module)	Baars et al (2005)^40^	To assess the HRQoL of children and adolescents with epilepsy and their families	10 items, 2 domains: Impact and Social	4‐16 y	Collaboration of seven European countries (Austria, France, Germany, Greece, the Netherlands, Sweden and the United Kingdom)	Child and parent report (parent proxy for 4‐7‐y olds)
9	Epilepsy and Learning Disability Quality of Life (ELDQoL)	Buck et al (2007)^41^	To assess HRQoL in children with both epilepsy and learning disabilities	70 items, 4 domains: Behaviour; Seizure severity; Mood and Side effects	2‐18 y	UK	Parent
10	Glasgow Epilepsy Outcome Scale (GEOS‐YP)	Townshend et al (2008)^42^	To assess the impact of epilepsy on an adolescent's QoL that is based on exploration of adolescent's views	50 items, 9 domains: Peer Acceptance; School/work; Development of Autonomy; Future focus; Epilepsy as part of Me; Medication issues; Seizures, Knowledge about Epilepsy; Sense of Uncertainty	10‐18 y	Glasgow, UK Tertiary epilepsy centres	Child
11	PedsQL Epilepsy Module	Follansbee‐Junger et al (2016)^44^	To validate a brief and reliable epilepsy‐specific, health‐related quality of life (HRQoL) measure in children with various seizure types, treatments, and demographic characteristics.	29 items, 5 domains: Impact; Cognitive; Sleep; Executive Function and Mood/behavior	2‐18 y	USA	Parent only report (2‐4‐y olds), and child and parent proxy report (aged 5‐18)

**Table 2:** Studies evaluating epilepsy‐specific patient reported outcome measures (PROMs)

Author	Instrument	Aim of study	Population	Mean age of children with epilepsy (SD)	Country	Funding
Hoare (1986)^22^	Adult's Attitudes to Children with Epilepsy: Visual Analogue Scale	To describe the use of a new visual analogue scale that compares attitudes to childhood epilepsy among parents and to assess its construct validity	Parents of children with epilepsy (n = 83), parents of children with diabetes (n = 26) and parents of children in healthy group (n = 50)	7.6 (3.8)	Edinburgh, UK	N/A
Hoare (1993)^21^	Modified Impact of Epilepsy Schedule	To assess the modified visual analogue scale on the assessment of QoL of children with epilepsy and their families and further assess its construct validity	108 school children with chronic epilepsy	10.4	Edinburgh, UK	N/A
Hoare and Russell (1995)^20^	The Impact of Childhood Illness Scale (ICI)	To develop prior Hoare scales to provide a more comprehensive assessment of the impact of epilepsy/long‐standing childhood illness on the QoL of the child and family and assess its content validity and construct validity	21 children aged 6‐17 y attending the epilepsy clinic	11 (2.9)	Edinburgh, UK	Small project grant from the Scottish Home and Health Department.
Hoare et al (2000)^19^	ICI	To assess the instrument on a group of children with epilepsy, assess the measures structural validity, internal consistency and to determine if the instrument can be applied to children with diabetes	102 children with epilepsy, 148 children with diabetes	9.6 (4.1)	Edinburgh, UK	N/A
Carpay et al (1997)^24^	The Hague Restrictions in Epilepsy Scale (HARCES)	To develop and assess the measures construct validity, internal consistency and test re‐test stability	122 parents of children with epilepsy (aged 4‐16) returned questionnaire	10.1 (3.3)	The Hague, Netherlands	Dutch National Epilepsy Fund, project number CLEOA 108.
Cramer et al (1999)^25^	Quality of Life in Epilepsy Inventory for Adolescents (QoLIE‐AD‐48)	To develop the QoLIE‐AD‐48 and assess its construct validity, structural validity, internal consistency, test‐retest reliability and proxy reliability	197 adolescents with epilepsy aged 11‐17 y	14.2 (1.7)	USA & Canada	N/A
Arunkumar et al (2000)^26^	Quality of Life in Pediatric Epilepsy (QoLPES)	To develop an instrument using parent‐ and patient‐validated content of quality of life measurement in children with epilepsy	80 parents and children Questionnaires completed by all 80 7 parents and 48 children themselves	4.5 (5.3)	USA	N/A
Sabaz et al (2000)^27^	Quality of Life in Childhood Epilepsy (QoLCE)	To develop the QoLCE and assess its internal consistency and construct validity	63 parents of children with refractory epilepsy	11	New South Wales, Australia	This work was supported by the National Health & Medical Research Council (NHMRC), Sydney Children's Hospital Foundation and Australian Brain Foundation.
Sabaz et al (2003)^28^	USQoLCE	To validate the QoLCE in an American population. To assess the content validity, internal consistency and construct validity of the US QoLCE	71 children	11	America	This research was supported by the National Health and Medical Research Council (NHMRC) and the Movement Disorder Foundation.
Talarska (2007)^29^	QoLCE	To assess the construct validity and reliability index of the QoLCE in a Polish population	160 children aged 8‐18 y and their parents	8‐18	Poland	N/A
Goodwin et al (2015)^30^	QoLCE‐55	To develop and validate a shortened version of the QoLCE by assessing the internal consistency, construct validity and structural validity.	373 children aged 4‐12 y and parents (data from HERQULES cohort)	7.5 (2.3)	Canada	This study was supported by a Canadian Institutes for Health Research operating grant (MOP‐64311)
Ferro et al (2016)^31^	QoLCE‐55	To examine the structural validity of the QoLCE‐55	373 children aged 4‐12 y and parents (data from HERQULES cohort)	7.5 (2.3)	Canada	Funded by a grant from the Canadian Institutes for Health Research (MOP‐64311)
Conway et al (2017)^32^	QoLCE‐55	To validate the newly developed shortened QoLCE‐55 in a sample of children with drug‐resistant epilepsy and asses its structural validity, internal consistency and construct validity	136 children aged 4‐18 enrolled in the Paediatric Epilepsy Surgery on HRQoL study (PEPSQoL)	11.5 (4)	Canada	Funded by a grant from the Canadian Institutes for Health Research (MOP‐64311)
Goodwin et al (2018)^33^	QoLCE 16	To develop a brief version of the QoLCE, assess its structural validity, internal consistency and construct validity	373 children and parents (data from HERQULES cohort)	7.5 (2.3)	Canada	PEPSQoL was funded by a grant from the Canadian Institutes for Health Research (MOP‐133708), HERQULES was funded by a grant from the Canadian Institutes for Health Research (MOP‐64311) to Conway is supported by a doctoral fellowship from the Social Sciences and Humanities Research Council.
Conway et al (2018)^34^	G‐QoLCE	To investigate the psychometric properties of a single item QoL measure the G‐QoLCE (responsiveness, construct validity, test‐retest reliability)	118 children with drug resistant epilepsy (PEPSQoL cohort)	11.5 (3.9)	Canada	PEPSQoL was funded by a grant from the Canadian Institutes for Health Research (MOP‐133708) LC is supported by a doctoral fellowship from the Social Sciences and Humanities Research Council.
Camfield et al (2001)^35^	Impact of Pediatric Epilepsy (IPES)	To assess the structural validity, construct validity, test‐retest reliability and internal consistency of the newly developed IPES	97 parents of children with epilepsy aged 2‐17 y	10.2 (4.5)	Canada	IWK Grace Research Foundation
Breau et al (2008)^36^	IPES	To assess the responsiveness of the IPES, 3 y after the IPES questionnaire was initially validated	97 parents/care‐takers of children with mild to severe epilepsy	10.7 (4.5)	Canada	N/A
Ronen et al (2001)^37^	Health‐Related Quality of Life Measure for Children with Epilepsy (CHEQoL‐25)	To use focus group methods to assess the different components of HRQoL in pre‐adolescent children with epilepsy. To form the development of the CHEQoL‐25 questionnaire	29 children with epilepsy aged 6‐12 y and their parents	6‐12 y	Canada	N/A
Ronen et al (2003)^38^	CHEQoL‐25	To evaluate the instruments construct validity, structural validity, assess test‐retest reliability and internal consistency of the CHEQoL‐25	381 children with epilepsy, aged 6‐15 y and their parents	10.8 (2.6)	Canada	Canadian Institutes of Health Research in partnership with GlaxoSmith‐Kline
Verhey et al (2009)^39^	CHEQoL‐25	To examine differences using the CHEQoL self‐report and parent proxy reports of QoL and examine its reliability	375 children and 378 parents independently completed data on the CHEQoL	6.9 (3.5)	Canada	Canadian Institutes of Health Research Health Professional Student Research Award.
Baars et al (2005)^40^	DISABKIDS (Epilepsy Module)	To describe the development of the condition‐specific modules of the European DISABKIDS (including the Epilepsy module) and evaluate its internal consistency and structural validity.	Children between 8‐16 y of age. 360 families, 37 participants with epilepsy	12.5 (2.5)	The project is a collaboration of seven European countries (Austria, France, Germany, Greece, the Netherlands, Sweden and the United Kingdom)	European Commission (QLG5‐CT‐2000‐00 716) within the Fifth Framework Program "Quality of Life and Management of Living Resources".
Buck et al (2007)^41^	Epilepsy and Learning Disability Quality of Life. (ELDQoL)	To describe the development of the ELDQoL and to assess the content validity, test re‐test reliability, internal consistency and construct validity of the measure.	Qualitative phase: 16 parents, 17 health professionals Psychometric phase: 47 parents/guardians, 21 formal carers of children with epilepsy	11.5 (4.6)	UK	N/A
Mcewan et al (2004)^43^	Glasgow Epilepsy Outcome Scale (GEOS‐YP)	To use focus group methods to investigate the impact of epilepsy on QoL of adolescents. To form the development of the GEOS‐YP scale	22 adolescents with epilepsy aged between 12‐18 stratified in to 6 focus groups.	14 y 1 mo	Scotland, UK	N/A
Townshend, et al (2008)^42^	Glasgow Epilepsy Outcome Scale (GEOS‐YP)	To develop and validate a measure of the impact of epilepsy on an adolescent and to assess its construct validity, internal consistency and test re‐test reliability	78 adolescents aged between 11‐18 y	15 (2.7)	Scotland, UK	N/A
Follansbee‐Junger et al (2016)^44^	PedsQL Epilepsy Module	To develop the PedsQL epilepsy module using focus groups and cognitive interviews	58 families. Children aged 5‐18 y diagnosed with epilepsy and their care‐giver. Care givers of children aged 2‐4 diagnosed with epilepsy also took part.	Focus group: 10.2 (4.5) Cognitive interview group: 8.91 (4.7)	USA	Fifth Third Bank/Charlotte R. Schmidlapp Women Scholars Program
Modi et al (2017)^45^	PedsQL Epilepsy Module	To assess construct validity, internal consistency, structural validity and test re‐test validity and measurement error of the PedsQL.	430 children with epilepsy and their care‐givers	9.9 (4.7)	USA	Charlotte Schmidlapp Women's Scholar Award, Cincinnati Children's Hospital Medical Center.
Sherman et al (2002)^23^	ICI, ICND, HARCES	To validate three measures of HRQoL in children with intractable epilepsy by assessing inter correlations.	44 children with intractable epilepsy	12 (3.8)	British Columbia's Children's Hospital, CA	British Columbia's Children's Hospital Foundation Telethon Competition New Research Fund and from the British Columbia Medical Services Foundation/Vancouver Foundation

**Table 3:** Methodological quality of studies evaluating measurement properties of PROMs using the COSMIN Risk of Bias checklist

Instrument & Author	1. PROM development	2. Content validity	Internal structure		5. Cross‐cultural validity/measurement invariance	6. Reliability	7. Measurement error	8. Criterion validity	9. Hypothesis testing for construct validity		10. Responsiveness
			3. Structural validity	4. Internal consistency		Test‐retest reliability			9a. comparison with other measure (convergent validity)	9b. comparison between subgroups (discriminative or known‐groups validity)	
Adult's attitude to children with epilepsy scale (Hoare, 1986)^22^										Doubtful	
Modified Impact of Epilepsy Schedule (Hoare, 1993)^21^										Doubtful	
Impact of Childhood Illness Scale (Hoare and Russell, 1995)^20^	Doubtful	Inadequate								Doubtful	
ICI (Hoare et al, 2000)^19^			Adequate	Very good						Doubtful	
HARCES (Carpay et al, 1997)^24^	Doubtful			Very good		Adequate				Adequate	
QoLIE‐AD‐48 (Cramer et al, 1999)^25^	Doubtful		Inadequate	Very good		Adequate			Adequate	Adequate	
QoLPES (Arunkumar et al, 2000)^26^	Adequate										
QoLCE (Sabaz et al, 2000)^27^	Doubtful			Very good					Adequate		
QoLCE (Sabaz et al, 2003)^28^		Doubtful		Very good					Adequate	Adequate	
QoLCE (Talarska, 2007)^29^				Doubtful						Inadequate	
QoLCE 55 (Goodwin et al, 2015)^30^			Very good	Very good					Very good		
QoLCE 55 (Ferro et al, 2016)^31^			Very good								
QoLCE 55 (Conway et al, 2017)^32^			Inadequate	Very good					Very good		
QoLCE 16 (Goodwin et al, 2018)^33^			Very good	Very good					Very good		
G‐QoLCE (Conway et al, 2018)^34^						Inadequate			Very good		Adequate
IPES (Camfield et al, 2001)^35^			Adequate	Very good		Doubtful			Doubtful	Adequate	
IPES (Breau et al, 2008)^36^											Doubtful
CHEQoL‐25 (Ronen et al, 2001)^37^	Adequate										
CHEQoL‐25 (Ronen et al, 2003)^38^			Very good	Very good		Doubtful				Very good	
CHEQoL‐25 (Verhey et al, 2009)^39^						aAdequate					
DISABKIDS (Baars et al, 2005)^40^	Adequate		Inadequate	Very good							
ELDQoL (Buck et al, 2007)^41^		Doubtful		Very good		Adequate			Adequate		
GEOS‐YP (Mcewan et al, 2004)^43^	Adequate										
GEOS‐YP (Townshend et al, 2008)^42^				Very good		Adequate			Very good	Very goo	
PedsQL Epilepsy Module (Follansbee‐Junger et al, 2016)^44^	Adequate										
PedsQL Epilepsy Module (Modi et al, 2017)^45^			Very good	Very good		Adequate	Adequate		Very good	Very good	
ICI, HARCES (Sherman et al, 2002)^23^									Adequate		

a Parent proxy reliability

**Table 4:** Summary appraisal of PROMs

Instrument version	Content validity	Structural validity	Construct validity	Internal consistency	Test‐retest reliability	Proxy reliability	Precision	Responsiveness
Adult's Attitude to Children with Illness Scale	0	0	?	0	0	0	0	0
Modified Impact of Epilepsy Schedule	0	0	?	0	0	0	0	0
Impact of Childhood Illness Scale (ICI)	?	+	+	++	0	0	0	0
Hague Restrictions in Childhood Epilepsy Scale (HARCES)	?	0	+	++	++	0	0	0
Quality of Life in Epilepsy inventory for Adolescents (QoLIE‐AD‐48)	?	+	+	+/−	++	0	0	0
Quality of Life in Paediatric Epilepsy (QoLPES)	+	0	0	0	0	0	0	0
Quality of Life in Childhood Epilepsy (QoLCE‐76)	?	0	+++	+++	0	0	0	0
QoLCE‐55	0	+++	+++	+++	0	0	0	0
QOCLE‐16	0	++	+	++	0	0	0	0
G‐QoLCE	0	0	+	0	?	0	0	+
Impact of Paediatric Epilepsy Scale (IPES)	0	+	+	++	?	0	0	?
Health‐Related Quality of Life Measure for Children with Epilepsy (CHEQoL‐25)	++	++	++	+/−	+/−	−	0	0
DISABKIDS (Epilepsy Module)	++	?	0	++	0	0	0	0
Epilepsy and Learning Disabilities Quality of Life (ELDQoL)	?	0	+	++	+	0	0	0
Glasgow Epilepsy Outcome Scale (GEOS‐YP)	++	0	++	+/−	+	0	0	0
PedsQL Epilepsy Module	+	+	++	+/−	+/−	0	+	0

Note

Indices for summarising the measurement properties

0, Not reported: no studies found that evaluate this measurement property

?, Not clearly determined: studies were rated poor methodological quality; results not considered robust

−, Evidence not in favour: studies were rated good or excellent methodological quality; results did not meet standard criteria for this property

+/−, Conflicting evidence: studies were rated fair, good, or excellent methodological quality; results did not consistently meet standard criteria for this property eg not for all domain scales

+, Some evidence in favour: studies were rated fair or good methodological quality; standard criteria were met for the property

++, Some good evidence in favour: studies were rated good or excellent methodological quality standard criteria were met or exceeded

+++, Good evidence in favour: studies were rated good or excellent methodological quality; standard criteria were exceeded, results have been replicated

Impact of Childhood Illness scale is a parent‐rated 30 item PROM.^19^, ^20^ The ICI evolved from the Adult's Attitudes to Children with Epilepsy Visual Analogue Scale and the Modified Impact of Epilepsy Schedule (Tables 1 and 2).^21^, ^22^ The evaluation of content validity of the ICI was rated as *doubtful/inadequate* due to poor description of development.^20^ However, ICI has evidence of good structural validity and the two domains (Frequency and Importance) have excellent internal consistency (*α* = 0.92‐0.94).^19^, ^23^ In addition, ICI has some evidence of construct validity, as demonstrated by a moderate correlation with the Hague Restrictions in Childhood Epilepsy (HARCES) PROM (*r* = .60).^19^, ^23^

HARCES is a parent‐rated 10‐item PROM (Tables 1 and 2).^23^ HARCES was developed by asking parents of children with epilepsy to list daily life activities limited by epilepsy, but the study rated as *doubtful* due to limited details about the development. HARCES demonstrates good internal consistency (*α* = 0.89) and test‐re‐test reliability (*r* ^2^ = .93). Construct validity was assessed by correlating HARCES scores and clinical variables but with few substantive findings.^23^, ^24^

Quality of life in Epilepsy Inventory for Adolescents (QoLIE‐AD‐48) is a 48‐item PROM (Tables 1 and 2).^25^ QoLIE‐AD‐48 items were devised based on a literature review, existing measures, and focus groups, but was rated as *doubtful* for its risk of bias because of limited description of the methods of development. Structural validity was assessed using factor analysis on 191 participants, but the method was rated *inadequate* due to an inadequate sample size. Supporting evidence was found for the internal consistency of the overall scale, and subscales met the standard criteria except the three‐item health perception scale (*α* = 0.52). Test‐retest reliability and construct validity were good (Table 4).

Quality of Life in Paediatric Epilepsy Scale (QoLPES) is a 20‐item PROM (Table 1 and 2). There are parallel child‐ and parent‐report versions. Scale items were devised following consultation with children and parents who were asked to list in order of importance their concerns, which were then aggregated by the study researchers; the study was rated methodologically *adequate*, providing evidence of content validity (Table 4).^26^

Quality of Life in Childhood Epilepsy (QoLCE) is a parent‐reported PROM. It has four questionnaire versions with different numbers of items: QoLCE‐76, QoLCE‐55, QoLCE‐16, and a single item G‐QoLCE (Table 1 and 2).^27^, ^28^, ^29^, ^30^, ^31^, ^32^, ^33^, ^34^ The original QoLCE‐76 was developed by a literature review and by adapting items from established instruments. A focus group of epilepsy patients and professionals reviewed the questionnaire for its content and clarity, but this was not described thoroughly, so was rated *doubtful* for its risk of bias (Table 4, Appendix S2). QoLCE‐76 has excellent internal consistency for the overall summary score and the subscales have good internal consistency.^28^ There is extensive evidence of construct validity from two studies, with one study demonstrating that the QoLCE‐76 correlated moderately to highly with similar scales on the established Child Health Questionnaire Parent Form (CHQ‐P50) (Table 4).^27^, ^28^

QoLCE‐55 is a shortened version of the QoLCE‐76 (Table 2).^30^, ^31^, ^32^ Structural validity was assessed in three studies using factor analysis. One study was rated as *inadequate* due to small sample size, but two other studies were rated as *very good*. QoLCE‐55 total score and the individual subscales were shown to have excellent internal consistency. Two studies provide support for the construct validity of the QoLCE‐55; one study found a strong correlation between subscales of the CHQ‐P50 with relevant subscales of the QoLCE‐55 and weaker correlations with dissimilar constructs.^30^, ^31^, ^32^ A further study indicated moderate to strong correlations with similar subscales of the KIDSCREEN‐27 and weak to moderate correlations with dissimilar subscales (Table 4, Appendix S2).^30^

QoLCE‐16 has good structural validity and excellent internal consistency across all scales (*a* = 0.75‐0.92) and for the overall scale (*α* = 0.90).^33^ Results that were reported previously using the QoLCE‐55 and QoLCE‐76 were comparable to those generated using the QoLCE‐16 model, providing evidence of construct validity (Table 4). The G‐QoLCE is an overall QoL single‐item question derived from QoLCE‐76 with evidence of good construct validity and responsiveness (Table 4).^34^

The Impact of Paediatric Epilepsy Scale (IPES) is an 11‐item parent‐report PROM (Table 1 and 2).^35^, ^36^ Item development has not been published. The PROM has good evidence of structural validity and internal consistency. There was some evidence for construct validity. The responsiveness study over a 3‐year time frame was rated methodologically *doubtful* due to risk of bias (Table 4).^35^

The Health‐Related Quality of Life Measure for Children with Epilepsy (CHEQoL) is a 25‐item PROM; the parent and child versions include some overlapping items but some differ.^37^, ^38^, ^39^ CHEQoL items were devised through qualitative research with children with epilepsy. CHEQoL has good structural validity, assessed by factor analyses run separately on the child and the parent instruments.^37^ Internal consistency was good for the four subscales in both the parent and child questionnaires. Test‐retest reliability for the child questionnaire was acceptable (ICC = 0.59‐0.69) in the 8‐ to 15‐year group but less so in the 6‐ to 7‐year‐olds, suggesting the scale is robust for 8‐ to 15‐year‐olds. Test‐retest reliability was adequate for the parent questionnaire; however, this study was rated *doubtful* due to a lack of detail about the stability of participants during the interim period. Construct validity of the CHEQoL was examined extensively.^38^ The different subscales demonstrated good to excellent discriminative validity between children with fewer or more health problems related to their epilepsy, with one exception being the *Secrecy* subscale. Reliability between child and parent report was generally poor, indicating that these should be interpreted separately.

DISABKIDS Epilepsy Module is a 10‐item PROM developed to supplement the DISABKIDS chronic generic module (Table 1 and 2).^40^ The PROM has a child and parent version for 8‐ to 16‐year‐olds and a parent proxy version for 4‐ to 7‐year‐olds. DISABKIDS Epilepsy Module was developed following extensive literature review, focus groups, and interviews carried out with patients with epilepsy and their parents. Structural validity was assessed by a factor analysis on 37 participants with epilepsy; the study was rated methodologically *inadequate* for risk of bias due to sample size. DISABKIDS Epilepsy Module showed excellent internal consistency for the Impact domain (*α* = 0.89) and adequate for Social domain (*α* = 0.77).

The Epilepsy and Learning Disabilities Quality of Life (ELDQoL) is a 70‐item, parent‐reported PROM (Table 1 and 2).^41^ ELDQoL was developed using interviews with parents and piloted, but this information was published only as a conference abstract and excluded due to limited details available. The content of ELDQoL was assessed in interviews with parents and health professionals, and the study was rated methodologically *doubtful* due to lack of details. ELDQoL shows excellent internal consistency across the four scales; test‐retest reliability for each subscale was high. There is evidence for construct validity.^41^

Glasgow Epilepsy Outcome Scale for Young People (GEOS‐YP) is a 50‐item, adolescent report PROM.^42^, ^43^ Items were devised from focus groups.^42^ GEOS‐YP was internally consistent for the total score and subscales. Test‐retest reliability was good, and the study was rated methodologically *adequate*. Construct validity was good and the GEOS‐YP total correlated highly with the QoLIE‐AD‐48 total. There were moderate correlations between other scales that measured similar constructs.

Paediatric Quality of Life Inventory (PedsQL) Epilepsy Module is a 29‐item PROM with parent and child versions and parent only for ages 2‐4 years.^44^, ^45^ PedsQL Epilepsy module was developed using focus groups.^44^, ^45^ There is evidence of structural validity and internal consistency for both child and parent report. Test‐retest reliability was moderate (ICC = 0.59‐0.83). Construct validity was demonstrated between similar scales and by discriminating known groups. Standard errors of measurement were reported across domains ranging from 7.59 (Cognitive) to 12.61 (Sleep) for parent‐report scales, and 8.44 (Impact) to 14.68 (Sleep) for child‐report scales.

## 4 DISCUSSION

Our review identified 11 epilepsy‐specific PROMs of children's HRQoL. In particular, the QoLCE‐55 questionnaire has good evidence of structural validity, construct validity, and internal consistency and this was replicated in other studies. CHEQoL is also a leading candidate, with good evidence of content validity, structural validity, and construct validity. Choosing between these two might be decided by QoLCE being only by parent‐reported questionnaire, whereas CHEQoL can capture both child‐ and/or parent‐reported HRQoL. Only 4 of the 11 PROMs had evidence to support content validity: CHEQoL, DISABKIDS epilepsy module, GEOS‐YP, and PedsQL epilepsy module. Content validity is considered to be the most important measurement property because it is essential that items measure what the PROM purports they measure and that they are comprehensible to the target population.^15^

Based on our results, no PROM met the standard criteria for all measurement properties, with evaluations on responsiveness and precision most lacking. Despite evidence lacking for some measurement properties, the HARCES, CHEQoL, QoLCE‐76, QoLCE‐55, QoLCE‐16, DISABKIDS Epilepsy Module, and GEOS‐YP have good evidence in favor of at least two measurement properties in papers rated *good* or *excellent* for their methodological quality. There is more evidence for the QoLCE questionnaires as considerably more research has been published evaluating them in comparison to other PROMs. It is important to remember this imbalance of research completed on the PROMs so that we do not dismiss a PROM that may be robust and useful but has not yet been fully validated and researched due to lack of resources.

Other reviews have also highlighted and evaluated the current condition‐specific and generic PROMs that are available for use in childhood epilepsy.^8^, ^9^, ^10^, ^11^, ^12^, ^13^, ^14^ One review identified that there were 13 epilepsy‐specific PROMs for children and assessed the content of the PROMs with reference to the World Health Organization (WHO) definitions.^9^ We identified nine of the same PROMs from this review. The previous PROMs that we did not include from the review were the Epilepsy Foundations of America Concerns Index (EFA), the Glasgow Epilepsy Outcome Scale (GEOS‐C), the Impact of Childhood Neurologic Disability Scale (ICNDS), and the Epilepsy and Children Questionnaire (ECQ).^46^, ^47^, ^48^, ^49^ We did not include the EFA and GEOS‐C because they are PROMs for adults. Some broader condition‐specific neurological PROMs may be useful in epilepsy such as the ICNDs; however, these broader instruments were not included in the scope of our review.^50^ The ECQ was validated in an Italian population. We also found two further childhood epilepsy‐specific PROMs that were the GEOS‐YP and PedsQL Epilepsy Module. Our review is an evaluation of the measurement properties of epilepsy‐specific PROMs for children and the only one to our knowledge that references the COSMIN methodology. Our review, in combination with information on the specific content of PROMs^9^, are key complementary resources that can aid clinicians and researchers in the selection of PROMs for a specific purpose.

We followed our peer‐reviewed and published protocol, which describes a proportionate and pragmatic approach to review PROMs for children with epilepsy. We did not register the review on PROSPERO; however, our protocol is publicly available.^17^ As outlined in our protocol, only one reviewer extracted evidence of measurement properties from the included studies (HC). Nevertheless, each paper was discussed extensively and appraised by two reviewers (HC and CM), and both were involved in assigning a summary rating while reviewing the papers. We also followed methodology for assessing risk of methodological bias advocated by COSMIN.^15^, ^17^ However, carrying out the appraisal using the most recent COSMIN checklists proved challenging at times. It was evident that many studies of PROMs, particularly those studies published more than 15 years previously, were not reported in sufficient detail. For example, ICI and HARCES studies provided such limited information on the development of the PROMs leading to harsh ratings of methodological risk of bias. It is vital that PROM developers take account of potential methodological risks of bias and report adequate details of how measurement properties are evaluated.

It is important that an evaluation of a child's HRQoL provides the opportunity for the child to rate their own HRQoL alongside their parent/carer. For children, HRQoL is primarily about their social life and activities and perhaps less about other factors that parents may deem important. Verhey et al^39^ found that agreement between parent proxy and child self‐reports show lower parent agreement on abstract domains of HRQoL, similar to the findings of other studies.^39^ There were three PROMs that had parent and child parallel versions, but only the CHEQoL assessed parent proxy reliability. The CHEQoL parent‐proxy measure should be used to complement to the child self‐report measure and provides an independent parent perspective of a child's HRQoL.^51^, ^52^, ^53^ However, there will be situations where children are not able to self‐report or may not want to complete a questionnaire, and in those instances parent‐report can be considered separately. The COSMIN RoB checklist does not include a box to rate the bias of proxy reliability in studies. We made a strategic decision to use the checklist for the general *reliability* box, as we thought parent‐proxy reliability was an important property to assess and report on in our context.

Depending on your viewpoint, a potential strength or limitation of our systematic review was our strict inclusion criteria. We included only studies that were published, peer‐reviewed evaluations of measurement properties of PROMs. Some experimental studies may report incidental data relating to measurement properties, even if not the purpose of that study. We limited the review to include only studies where English‐language versions of PROMs were administered. This led to the exclusion of the ECQ developed in Italy, which showed promise for being a valid and reliable PROM in an Italian population of children.^46^ However, we cannot assume that measurement properties of PROMs are generalizable across different languages or cultures, which is a valid reason to exclude non–English‐language versions of PROMs from this review. Our study focused on English‐language versions of PROMs due to the objectives of the CASTLE research program, of which this work is a part of, and to inform trial design. However, we also recognize that research operates on an international level, and there will need to be reviews of evidence of validity and reliability when evaluated in languages other than English. We also included the ELDQoL, although it has a slightly different focus as it is for children with both epilepsy and learning disability. We included this PROM in the scope of our review because the questions are focused on aspects of epilepsy but asked in a way that is more appropriate to parents of children with a learning disability. However, we did not include the Paediatric Refractory Epilepsy Questionnaire that was found in our search, as this would not be appropriate for all children with epilepsy.^50^

For clinicians and researchers to make an informed decision about which PROM to choose for a specific objective, there are other important properties to consider such as what constructs of HRQoL they measure, their importance to children with epilepsy and families, and how the questions and response options are understood by respondents. It is crucial to consider other important factors such as the content of a PROM and its face validity and acceptability to respondents. In a related paper,^54^ we examine the content of each PROM from this review and map the individual questions to our recently developed core outcome set for childhood epilepsy research.^55^ In addition, we report on our consultations with families of children with epilepsy about the acceptability and practicalities of using the QoLCE and CHEQoL.

In conclusion, from the evidence we have synthesized there are a small number of epilepsy‐specific PROMs of children's HRQoL with enough evidence of robust measurement properties to recommend them. In particular, the QoLCE‐55 and CHEQoL are leading candidates, of which only CHEQoL offers child self‐reported HRQoL. It is also evident that parent proxy report is not always a reliable way of assessing subjective HRQoL in children, and parent and child reports collected separately is the best way of ensuring that the child's HRQoL is assessed. There remains a pressing need for research to evaluate the responsiveness of these measures so we can interpret what constitutes meaningful change in scores over and above measurement error.

## CONFLICTS OF INTEREST

The authors declare that they have no competing interests. We confirm that we have read the Journal's position on issues involved in ethical publication and affirm that this report is consistent with those guidelines.
