# Supplementary material for: Epilepsy‐specific patient‐reported outcome measures of children's health‐related quality of life: A systematic review of measurement properties
Source: Epilepsia. 2020 Jan 17;61(2):230–48. doi: 10.1111/epi.16430 (PMC7065094; doi:10.1111/epi.16430)
Supplement: Supplementary file 1 [file EPI-61-230-s001.docx]

MEDLINE search strategy

Database: Ovid MEDLINE(R) Epub Ahead of Print, In-Process & Other Non-Indexed Citations, Ovid MEDLINE(R) Daily and Ovid MEDLINE(R) <1946 to Present>

Search Strategy:

--------------------------------------------------------------------------------

1 disabkids.ti. and epilepsy.ti,ab. (2)

2 "epilepsy and children questionnaire".ti. (0)

3 ECQ.ti. and epilepsy.ti,ab. (0)

4 "Epilepsy and learning disabilities quality of life".ti. (1)

5 "Epilepsy foundations of america concerns index".ti. (0)

6 "Glasgow epilepsy outcome scale".ti. (4)

7 ELDQOL.ti,ab. (7)

8 EFA.ti. and epilepsy.ti,ab. (0)

9 GEOS C.ti,ab. (1)

10 "hague restrictions in childhood epilepsy".ti. (0)

11 HARCES.ti,ab. (6)

12 CHEQOL.ti,ab. (11)

13 "impact of childhood illness".ti. (2)

14 (ICI and epilepsy).ti. (0)

15 "Impact of Childhood Neurologic Disability".ti. (1)

16 ICNDS.ti,ab. (3)

17 "Impact of Pediatric Epilepsy".ti. (9)

18 (IPES and epilepsy).ti,ab. (11)

19 QOLCE.ti,ab. (46)

20 QOLIE.ti. (41)

21 "Quality of Life in Epilepsy Inventory".ti. (29)

22 "Quality of Life in Pediatric Epilepsy".ti. (6)

23 QOLPES.ti,ab. (0)

24 CHQ 50.ti,ab. (4)

25 child health questionnaire.ti. and epilepsy.ti,ab. (3)

26 "pediatric quality of life inventory".ti. and epilepsy.ti,ab. (1)

27 PEDSQL.ti. and epilepsy.ti,ab. (2)

28 "Quality of Life in Neurological disorders pediatric version".ti. (0)

29 pednqol.ti,ab. (0)

30 or/1-29 (155)

31 epilep*.ti. (73389)

32 (measure* or tool* or questionnaire* or scale* or inventor*).ti. (440722)

33 (child* or pediatric or paediatric or adolesc* or infant*).ti. (1072774)

34 "quality of life".ti. (58618)

35 31 and 32 and 33 (131)

36 31 and 33 and 34 (184)

37 31 and 32 and 34 (108)

38 epilepsy.ab. (71807)

39 32 and 33 and 34 and 38 (37)

40 30 or 35 or 36 or 37 or 39 (409)
