# Supplementary material for: Epilepsy‐specific patient‐reported outcome measures of children's health‐related quality of life: A systematic review of measurement properties
Source: Epilepsia. 2020 Jan 17;61(2):230–48. doi: 10.1111/epi.16430 (PMC7065094; doi:10.1111/epi.16430)
Supplement: Supplementary file 2 [file EPI-61-230-s002.docx]

**Narrative synthesis of data extracted on measurement properties for each PROM**

**1. The Impact of Childhood Illness Scale (ICI)**^19^

This Impact of Childhood Illness (ICI) scale is a parent rated 30-item PROM with 4 domains. The purpose of the instrument is to assess the impact of epilepsy/long-standing illness on quality of life (QoL) on the child (aged 6-17 years) and family. It is scored using two dimensions; ‘Frequency’ and ‘Importance’. The ICI has been developed from two prior scales; the Adult’s Attitudes to Children with Epilepsy Visual Analogue Scale and the Modified Impact of Epilepsy Schedule.^19,20,21,22^ The construct validity was assessed in the two prior versions, but this was rated as *doubtful* for its risk of bias.

The instrument was later re-named the ICI in a paper that describes the development, content validity and construct validity of the PROM but all properties were rated as *doubtful/inadequate* in this paper for risk of bias due to limited description about the development.^20^ Two further papers assessed the instruments structural validity, internal consistency and construct validity.^20,21^ The structural validity was assessed using factor analysis (FA) on 250 participants which showed there was one main factor, ‘impact on the parents and the family’ in a study rated *adequate* for its risk of bias*.* Internal consistency for the measure was excellent, Cronbach’s α = 0.94 for the Frequency dimension and α = 0.92 for the Importance dimension. It was rated as *very good* for risk of bias for internal consistency. Construct validity was demonstrated as the ICI was discriminant between children with epilepsy and diabetes. The ICI was found to have a moderate correlation with the HARCES scale as assessed by Spearman’s *r* (0.60) and the study was rated as *adequate* for risk of bias.^23^

**2. Hague Restrictions in Childhood Epilepsy Scale (HARCES)**^24^

The HARCES is a parent rated 10-item instrument that quantifies restrictions due to disability in children aged 4-16 years with epilepsy. The instrument is evaluated in two studies which assess its development, construct validity, internal consistency and test re-test stability.^23,24^ The instrument was developed by asking parents of children with epilepsy to list their daily life activities that were limited by their child’s epilepsy. The items were reviewed by participating neurologists however the study is rated as *doubtful* for its risk of bias. The HARCES has excellent internal consistency (α = 0.89) in a study rated as *very good* for risk of bias. Test re-test reliability is excellent (r^2^ =0.93) in a study rated as *adequate* for risk of bias. Construct validity was assessed by looking at the association between the HARCES scores and a priori clinical variables. When the neurologist had advised imposing restrictions, the median score was higher than when this was not advised. No other substantial correlations were found. The study was rated as *adequate* for risk of bias. In a second study, the HARCES correlated moderately with the ICI (α = 0.60), and this study was rated as ‘adequate’ for risk of bias.^22^

**3. Quality of Life in Epilepsy Inventory for Adolescents (QOLIE-AD-48)**^25^

The QOLIE-AD-48 is an adolescent rated, 48-item instrument with 8 domains. The purpose of the instrument is to assess the HRQoL in adolescents aged 11-17 years with epilepsy. One study has evaluated the QOLIE-AD-48 assessing its development, structural validity, internal consistency, test re-test reliability and construct validity.^7^ The instrument items were developed based on a literature review, existing measures and a focus groups in a study rated as *doubtful* for its risk of bias. Structural validity was assessed using factor analysis on 191 participants, but the method was rated as *inadequate* for risk of bias due to inadequate sample size. Supporting evidence was found for the internal consistency of the overall scale as assessed by Cronbach’s alpha (α =0.74) and subscales met the standard of (α=>0.7) except the three-item health perception scale (α =0.52). The risk of bias for internal consistency was rated as *very good*. The test-retest reliability was excellent as measured by an Intraclass Correlation coefficient (ICC=0.83) and rated *adequate* for risk of bias.

Supporting evidence was provided for construct validity of the QOLIE-AD-48 by examining the association between the QOLIE-AD-48 with the Rosenberg Self-Esteem Scale (RSE; 20), the Ilfeld Self-Efficacy Scale (ISE:21) and a 11-item parent questionnaire. The self-esteem and self-efficacy scales were hypothesized to correlate with the HRQOL constructs in a study rated *adequate* for risk of bias. The overall summary scale correlated moderately with the self-esteem scale (*r* = 0.65); self-efficacy scale (*r*=0.54). In addition, the parental questionnaire correlated well with the summary score of the QOLIE-Ad-48 (*r*= 0.67), indicating similar perceptions between parents and adolescents. It was also hypothesised that adolescents having fewer and less sever seizures would have better HRQoL than those with more frequent and severe seizures. The scale discriminated well between different categories of seizure frequency as QOLIE-AD-48 scores decreased (worsened) as seizure severity increased.

**4. Quality of Life In Paediatric Epilepsy Scale (QOLPES)**^26^

The QOLPES is a 20-item instrument that assesses the HRQoL in children with epilepsy aged 3 months to 18 years. There are two parallel versions, a parent report and child report instrument. One study describes the instrument’s item development.^8^ The items of the scale were devised following consultation from children and parents who were asked to list in order of importance their concerns which were aggregated by the study researchers. The study was rated as *adequate* in the risk of bias checklist for development. We found no further evaluations of this instrument.

**5a. Quality of Life in Childhood Epilepsy (QOLCE)**^27,28^

The QOLCE is a parent report, 76-item instrument that has been evaluated in three studies.^27-29^ The purpose of the QOLCE is to assess the HRQoL of children with epilepsy. Initially, the instrument was developed in Australia in a study that assessed the instruments internal consistency and construct validity.^27^ It was then validated in the US in a study that assessed the instruments internal consistency and construct validity.^28^ Five further studies assessed shortened versions of the QOLCE-76; a 55-item scale, a 16-item scale and a single item scale.^30-34.^

***QOLCE-76***^27,28^

The QOLCE was developed by literature review, adaptation of items from established instruments and written responses to a qualitative questionnaire by patients and their guardians. A focus group of epilepsy patients and professionals reviewed the questionnaire for its content and clarity, but this was not described thoroughly. Subsequently, the studies were rated as *doubtful* for risk of bias for development and content validity. The QOLCE has excellent internal consistency for the overall summary score as demonstrated in three studies (α =0.92-0.93) and the multi item scales have good internal consistency as reported in two studies (α =0.69-0.97). The risk of bias rating for two of the studies for internal consistency was *very good*.

Construct validity was assessed by comparing the QOLCE to the Child Health Questionnaire (CHQ).^10^ Correlations between similar scales were moderate to high in value (r=0.54-0.75). Correlations between dissimilar constructs should be low relative to the convergent validity, and this was the case for some but not all of the constructs (r=0.17-0.49). The QOLCE was compared to the Child Behaviour Checklist (CBCL), and the QOLCE attention/concentration scale correlated modestly to the attention problems scale on the CBCL (r=-0.67). Specifically, as parent-reported attention increased in the epilepsy-specific scale, there were fewer reported problems with attention recorded by the CBCL. This study was rated as *adequate* for risk of bias for construct validity.

Construct validity was further assessed by comparing the QOLCE to the CHQ.^9^ The QOLCE correlated moderately to highly with theoretically similar subscales on the CHQ (r=0.46-0.70). The remaining correlation coefficients for dissimilar scales were generally lower than the convergent validity (r=0.17-0.58). Inpatients had significantly lower QOLCE scores compared with outpatients, and seizure severity over the past 6 months had a negative relationship with QOLCE subscales except for the energy/fatigue, depression, social interaction, and behaviour subscales. Children with >90% of reduction in seizure frequency had higher overall QOLCE score compared to those with <90% reduction in seizures. This study was rated as *adequate* for risk of bias for construct validity.

***5b. QOLCE-55***^30^

Three studies provide support for the QOLCE-55 which assess the instruments structural validity, internal consistency, and construct validity.^30-34^ Structural validity was assessed by all three studies using factor analysis. One study was rated as *inadequate* for risk of bias due to a small sample size, but two other studies were rated as *very good*. The QOLCE-55 is internally consistent as assessed by Cronbach’s alpha from two studies which was excellent (α = >.80) for the total score and the individual subscales. The risk of bias rating for internal consistency across the studies was *very good*.

Two studies provide support for construct validity of the QOLCE-55, rated as *very good* for risk of bias. One study (Goodwin et al., 2015) examined the correlation of relevant subscales of the CHQ with relevant subscales of the QOLCE-55 using Spearman’s rho (p). Correlations were found to be moderate to strong ranging from 0.38-0.70. For divergent validity, correlations examined were weak (p=0.30-0.31). A further study (Conway et al., 2017) demonstrated that there were moderate to strong correlations with similar subscales of the KIDSCREEN-27 and QOLCE-55 using Spearman’s rho (p=0.43-0.75) and weak to moderate correlations with dissimilar subscales QOLCE-55 (p=0.25-0.42).

***5c. QOLCE-16***^33^

The QOLCE-16 is an instrument developed from the QOLCE-55 and its structural validity, internal consistency and construct validity is assessed in one study.^33^ The QOLCE-16 has good structural validity as assessed by a factor analysis and rated as *very good* for risk of bias. The QOLCE-16 has excellent internal consistency as measured by Cronbach’s alpha (α = >0.7 for total score and individual scales) and the risk of bias was rated as *very good*. Results that were reported previously using the QOLCE-55 and QOLCE-76 were comparable to those generated using the QOLCE-16 providing evidence of construct validity and rated as *adequate* for risk of bias.

***5d. G-QOLCE***^34^

The G-QOLCE is an overall QOL question derived from the QOLCE-76 instrument. One study provides support for the instruments test-retest reliability, construct validity and responsiveness.^34^ The test-retest reliability was assessed with an ICC from baseline to 6-months. The ICC for the composite score is 0.72. ICC values for subgroups of clinically stable patients are considered fair to good (ICC= 0.49–0.72). The study was rated as *inadequate* for risk of bias as the time interval was not typically appropriate.

The construct validity was examined by measuring the correlation of the G-QOLCE with the QOLCE-76 composite score (excluding the QOL item) and five main domains, as well as KIDSCREEN-27 composite score and five subscales, at baseline and 6-month follow-up. The majority of QOLCE-76 domains and KIDSCREEN-27 subscales are moderately correlated with G-QOLCE scores. The G- QOLCE scores are moderately correlated with composite scores of the QOLCE-76 (ρ=0.54) and main domains of physical activity (6 months) (ρ=0.48) and social activity (6 months) (ρ=0.49), and with the KIDSCREEN-27 composite scores (ρ=0.52) and the physical well-being (ρ=0.50) and psychological well-being subscales (ρ=0.52). The strength of the correlations increased over time, with the exception of QOLCE-76 well-being domain (ρ=0.38). The risk of bias rating for construct validity was *very good*.

To assess clinically relevant change, responsiveness was examined through the distribution-based and anchor-based methods. Using distribution-based methods, the G-QOLCE is able to detect changes in patient QOL. For anchor-based-methods, G-QOLCE scores from baseline to 6-month follow-up demonstrated a moderate magnitude of change for patients who “changed” based on the composite scores of the QOLCE-76 excluding the QOL item and the KIDSCREEN-27. The risk of bias for responsiveness was rated as *adequate.*

**6. Impact of Paediatric Epilepsy Scale (IPES)**^35^

The IPES is an 11-item parent report instrument. The purpose of the instrument is to assess the influence of epilepsy on the major aspects of the family and child’s life in children aged 2-16 years with epilepsy. There are two studies that provide support for the IPES which assess the instruments structural validity, internal consistency, test-retest reliability, construct validity and responsiveness.^35,36^ The items for the IPES are developed from prior work but this study has not been published. Structural validity was assessed by factor analysis in a study rated as *adequate* for its risk of bias. The scale has excellent internal consistency as assessed by Cronbach’s alpha (α = 0.92) for the first administration of the IPES and (α = 0.94) for the second. The study was rated as ‘very good’ on the risk of bias checklist. The test-retest reliability of the total score was assessed using Pearson’s *r*, (r=0.81), but an ICC was not calculated which led to a risk of bias rating as *doubtful* for this study.

The construct validity was assessed by comparing the individual scores of the IPES with other scales (a companion questionnaire that measured the same attributes, Family Environment Scale, Parenting Stress Index, Visual Analogue Scale (VAS), Pier-Harris Children’s Self-Concept Scale, Brother-Sister Questionnaire, Loneliness Scale and the Academic Performance Rating Scale) and within sub groups. There was no correlation between the total scores with the other scales except for a moderate negative correlation with total score and VAS rating. Children with low and high impact scores were compared and children with high impact scores had more clinical problems (behaviour, cognitive, neurological), more medication use and increased physician visits than the low impact group. This study was rated as *doubtful* for construct validity between other measures and *adequate* for comparison between subgroups. The IPES is responsive to changes in children’s epilepsy severity over a 3-year time frame: when epilepsy severity changed, the IPES responded accordingly. The study was rated as *doubtful* for risk of bias for responsiveness.

**7. Health-Related Quality of Life Measure for Children with Epilepsy (CHEQOL)**^39^

The CHEQOL is a 25-item instrument with parallel parent and child versions (6-15 years). The purpose of the instrument is to measure the HRQOL of pre-adolescent children with epilepsy. There are three studies that provide support for the instrument that assess its development, structural validity, internal consistency, test re-test reliability, proxy reliability and construct validity.^28-40^ The instrument is developed from modified focus group techniques in children with epilepsy to develop which later became the items for the instrument. The study is rated as *very good* for its risk of bias.^38^

The CHEQOL has good structural validity, assessed by factor analyses run separately on the child and the parent instrument. There were 5 factors on the child report instrument and 4 for the parent The study was rated as *very good* for the its risk of bias. Internal consistency was good (a = >0.70) for the four subscales in both the parent and child instrument, a slight lower value of a = 0.63 for the subscale *Quest for Normality* in the child scale, and a=0.64 for the *Present Worries* subscale in the parent instrument. The internal consistency was rated as *very good* in this study for risk of bias. The test-retest reliability for the child instrument was acceptable (ICC= 0.59 – 0.69) but only in the 8-15-years group, children in the 6-7-years group had more unreliable ICC’s. This suggests the scale is psychometrically robust for 8-15-year olds. The test-retest reliability was adequate for the parent instrument (ICC = 0.60-0.81). The internal consistency was rated as *very good* in this study for risk of bias.

The construct validity of the CHEQOL has been explored extensively. The different subscales showed good to excellent discriminative validity between children with fewer or more health problems related to their epilepsy, with one exception being the *Secrecy* subscale. The child’s self-report instrument showed slightly better discriminative properties that the parent report instrument. The study was rated as *very good* for its risk of bias.

CHEQOL was assessed for its proxy reliability. It was hypothesised that more concrete, external domains of HRQoL such as *interpersonal-social consequences* of epilepsy correlated more highly between parent and child report, whereas less visible abstract domains such as secrecy. Agreement between child and parent ranged from ICC= 0.24-0.49. Parents rated the HRQoL of the child than did the children themselves on the *Total Score*. Discordance for test re-test reliability was for the *secrecy* subscale (ICC =0.24) where the children’s rating was higher than the parents, and *Present Concerns* (ICC=0.32) where the child’s rating was lower than the parents. Children and parents agreed more on the external domains. There is no COSMIN box for ‘proxy reliability’ therefore we have rated the study as ‘*adequate*’ for reliability with a note that it also measures this psychometric property well.

**8. DISABKID Epilepsy Module**^41^

The DISABKIDS Epilepsy Module is a 10-item instrument with 2 domains (Impact and Social) which has been developed as a supplement to the chronic generic module. The aim of the instrument is to assess the HRQoL of children and adolescents with epilepsy. The instrument has a child and parent version, for children aged 8-16-years and a parent proxy version for children aged 4-7 years old. One study provides evidence for the instrument’s development, structural validity and internal consistency for the adolescent reported instrument.^23^ The DISABKIDS epilepsy module is developed following a stepwise methodology of questionnaire construction which include an extensive literature review and focus group and interviews carried out with patients with epilepsy and their parents, the risk of bias for this property was rated as *adequate*. Structural validity was assessed by a factor analysis on 37 participants with epilepsy, the study is rated as *inadequate* for its risk of bias for this property due to an inadequate sample size. The DISABKIDS has excellent internal consistency for its Impact domain (a=0.89) and Social domain (a=0.77) in the adolescent version, and the study is rated as *very good* for its risk of bias.

**9. Epilepsy and Learning Disability Quality of Life**^42^

The ELDQOL is a 70-item, parent report instrument with 4 domains and it is parent report. The purpose of the instrument is to assess the HRQoL in children with both epilepsy and learning difficulties. There is one study that provides evidence for the instruments content validity, internal consistency, test-retest reliability and construct validity.^42^ The instrument is developed based on in-depth interview with parents of children with severe epilepsy and piloted, but this this information was published as a conference abstract and was therefore excluded from the literature review accessed. The PROM was revised and reassessed for its measurement properties in a further study. The content of ELDQOL was assessed with qualitative interviews with parents and health professionals to ascertain opinions on wording, the study is rated as *doubtful* for risk of bias due to the lack of information.

The instrument has excellent internal consistency across the four scales as assessed by Cronbach’s alpha (0.74-0.95) in a study that is rated *very good* for risk of bias. The test-retest reliability for each subscale is high (ICC= 0.80-0.96) but the study is rated as *adequate* for risk of bias. Evidence of construct validity is good, all but one of the four ELDQOL subscales (behaviour) is moderately to highly correlated with both the Irritability and Hyperactivity scales of the Aberrant Behaviour Checklist (ABC), and all were moderately to highly correlated with the Emotional Impact and Family Activities Scales of the Child Health Questionnaire (CHQ). Poorer perceived health is related to poorer mean scores on two of the ELDQOL subscales. Construct validity is rated as *adequate* for risk of bias.

**10. Glasgow Epilepsy Outcome Scale for Young People**^43^

The GEOS-YP is a 50-item, adolescent report instrument that is split in to two parts; Identity Formation (part 1) and Epilepsy-Related Issues (part 2). The purpose of the instrument is to assess the impact of epilepsy on an adolescents QoL. There are two studies that provide evidence for the instrument’s development, internal consistency, test-retest reliability and construct validity.^43-44^ The items for the GEOS-YP are developed from focus group methods in adolescents with epilepsy in a study that is rated as *very good* for its risk of bias.^26^ The GEOS-YP is internally consistency for the total score (a = 0.91), Part 1 (a=0.86) and Part 2 (a=0.81). Subscale values ranged from a=0.49-0.82. The study was rated as *adequate* for risk of bias for this property. The test-retest reliability was good (p=0.75) and the study was rated *adequate* for risk of bias. The construct validity was good, the GEOS-YP total correlated highly with the QOLIE-AD-48 total (r=0.77). There were moderate correlations between subscales that measured similar constructs. The GEOS-YP correlated with the PedsQL total (a=0.74) and other strong correlations were found between similar subscales. The GEOS-YP correlated inversely to the Rosenburg self-esteem scale (RSE) (r=-0.66). It was also found that that GEOS-YP total scores were higher for those that were seizure free compared to those with a high seizure frequency. Participants on polytherapy scored lower than participants on monotherapy, suggesting further discriminant validity. The study was rated as ‘very good’ for risk of bias.

**11. Paediatric Quality of Life Inventory (PedsQL) Epilepsy Module**^45^

The PedsQL Epilepsy Module is a 29-item instrument split in to 5 domains. It has parallel parent and child versions for ages 5-18 (Young Child, Child, and Teen subgroups) and parent report only for Toddlers aged 2-4 years. The aim of the instrument is to assess the HRQoL of young people with epilepsy. There are two studies that provide evidence for its development, structural validity, internal consistency, test-retest reliability, construct validity and measurement error.^45-46^

The PedsQL Epilepsy module was developed by qualitative focus groups with young people with epilepsy and their parents, in a study rated *adequate* for risk of bias.^45^ Structural validity was assessed by a factor analysis and was rated as *very good* for risk of bias. Internal consistency coefficients for each scale are strong for both child and parent report (a=0.64-0.95) with the lowest alpha for the Sleep Scale in 2-4-year old children. Test re-test reliability was moderate ranging from ICC=0.59-0.83. Internal consistency was rated as *very good* for its risk of bias and test-retest reliability was rated as *adequate*. Construct validity was extensively explored, strong correlations were found between similar scales, especially for parent reports. Total side effects were correlated with all parent report and child report Epilepsy PedsQL Module scales. The PedsQL Epilepsy module has good divergent validity, for example children on monotherapy had higher HRQoL compared to children on polytherapy across both parent and child reported PedsQL Epilepsy Module scales. Risk of bias for content validity was rated as *very good* for this study. The PedsQL epilepsy module had good precision, standard errors of measurement ranging from 7.59 (Cognitive) to 12.61 (Sleep) for parent-report scales and 8.44 (Impact) to 14.68 (Sleep) for child-report scales. The study is rated as *adequate* for measurement error on risk of bias.

**References – see manuscript**
